# Education-only versus a multifaceted intervention for improving assessment of rehabilitation needs after stroke; a cluster randomised trial

**DOI:** 10.1186/s13012-016-0487-2

**Published:** 2016-09-07

**Authors:** Elizabeth A. Lynch, Dominique A. Cadilhac, Julie A. Luker, Susan L. Hillier

**Affiliations:** 1 Department of Health Sciences, International Centre for Allied Health Evidence, University of South Australia, GPO Box 2471, Adelaide, 5001 Australia; 2 Stroke Division, The Florey Institute of Neuroscience and Mental Health, 245 Burgundy St, Heidelberg, 3084 VIC Australia; 3 NHMRC Centre of Research Excellence in Stroke Rehabilitation and Brain Recovery, Parkville, VIC Australia; 4 Department of Medicine, Stroke and Ageing Research Centre, Monash University, Clayton, 3800 VIC Australia

## Abstract

**Background:**

In 2011, more than half of the patients with stroke in Australian hospitals were not assessed for the need for rehabilitation. Further, there were no recommended criteria to guide rehabilitation assessment decisions. Subsequently, a decision-making tool called the Assessment for Rehabilitation Tool (ART) was developed. The ART was designed to assist Australian hospital clinicians to identify the rehabilitation needs of patients with stroke using evidence-based criteria. The ART was released and made freely available for use in 2012. This study evaluated the effectiveness of an education-only intervention (1 onsite education session and distribution of the ART) and a multifaceted intervention (2 or more onsite education sessions, distribution of the ART, audit and feedback, barrier identification, site-specific strategy development, promotion of interdisciplinary teamwork, opinion leaders and reminders) for improving assessments of rehabilitation needs after stroke.

**Methods:**

Ten hospitals in 2 states of Australia were randomly assigned to an education-only or a multifaceted intervention. Medical records were audited by assessors blinded to group allocation before and after the implementation period. Difference in the proportion of patients assessed for rehabilitation before and after the intervention was analysed using mixed-effects logistic regression analysis, with time period as the dependent variable, an interaction between intervention type and time included to test for differences between the interventions, and hospital included as the random effect to account for patient clustering.

**Results:**

Data from 586 patients (284 pre-intervention; 302 post-intervention; age 76 years, 59 % male) showed that the multifaceted intervention was not more effective than education-only in improving the proportion of patients whose rehabilitation needs were assessed (reference category education-only; odds ratio 1.29, 95 % confidence interval 0.63–2.67, *p* = 0.483). Post-intervention, the odds of a patient’s rehabilitation needs being assessed was 3.69 times greater than pre-intervention (95 % confidence interval 2.57–5.30, *p* < 0.001). Evidence-based criteria were not consistently used when patients were deemed to have no rehabilitation needs.

**Conclusions:**

A multifaceted intervention was not more effective than education-only in improving the assessment of rehabilitation needs of patients with stroke. Further interventions are required to ensure that all patients are assessed for the need for rehabilitation using evidence-based criteria.

**Trial registration:**

ANZCTR (Australian New Zealand Clinical Trials Registry), ACTRN12616000340437

**Electronic supplementary material:**

The online version of this article (doi:10.1186/s13012-016-0487-2) contains supplementary material, which is available to authorized users.

## Background

Stroke is the second most common cause of death and the third most common cause of disability-adjusted life years worldwide [1]. Rehabilitation after stroke reduces death and disability [2]. Rehabilitation needs after stroke will not always be identified without a specific assessment for rehabilitation [3]. Accordingly, Australian clinical guidelines include a recommendation that every patient with stroke, who is not receiving palliative care, should be assessed for the need for further rehabilitation before leaving hospital [4]. However, in 2011 and 2013, less than half of the patients with stroke in Australian hospitals were assessed for ongoing rehabilitation needs [National Stroke Foundation, unpublished data].

In the immediate post-stroke phase, stroke unit care leads to better patient outcomes [5]. One of the defining features of a stroke unit is coordinated care provided by a multidisciplinary team (medical, nursing and allied health professionals) with expertise in stroke. In many developed countries (for example the UK, USA, and Australia) the median length of stay in the acute hospital after stroke is less than 7 days [6–8]. Therefore, a referral to a rehabilitation service is required if a person with stroke requires rehabilitation beyond this time. Rehabilitation can be provided in a person’s home, in the community or in inpatient settings.

Published information is lacking about the criteria used by hospital clinicians to determine the rehabilitation needs of patients with stroke. Patients with severe stroke in Australia are not always referred for ongoing rehabilitation [9]. Similarly, patients with mild stroke tend not to be referred to rehabilitation services despite frequently experiencing ongoing difficulties with community mobility, return to leisure and work activities, or having altered cognition or mood [10, 11]. It is important that staff working with patients with stroke accurately and consistently assess patients’ rehabilitation needs in order to identify patients who require referrals to rehabilitation services.

Data are collected in acute Australian hospitals biennially to audit the delivery of best practice stroke care [12]. Medical records of patients with stroke are audited by hospital clinicians, and site-specific and national reports are prepared by the National Stroke Foundation. One question included in the audit is whether the patient had a formal assessment for inpatient rehabilitation. To address concerns regarding the low proportions of patients assessed for rehabilitation, and variability in rehabilitation assessment and referral processes [13, 14], the Australian Stroke Coalition (an alliance of organisations and groups working in the stroke field) developed the Assessment for Rehabilitation Pathway and Decision-Making Tool (ART) in 2011 [15]. The ART was developed collaboratively by a multidisciplinary working group and consultation with stakeholders following a systematic review of the literature. The ART was designed to help health professionals make objective decisions regarding the ongoing rehabilitation needs of every patient with stroke admitted to hospital. Based on the best available evidence, the default position of the ART was that every patient with stroke would benefit from rehabilitation unless one of four pragmatic exclusion criteria is met: the patient makes a full recovery; refuses rehabilitation; is persistently non-responsive; or is receiving palliative care. Every person with stroke who does not meet the ART exception criteria should be referred to a rehabilitation service to determine whether the patient’s rehabilitation needs can be met. In this way, use of the ART could help to quantify unmet rehabilitation needs of patients with stroke.

The ART was piloted in 2011 in six Australian states. The process of developing the ART (literature review, involvement of key stakeholder groups) ensured its content validity. During the pilot testing phase, the ART was found to have good face validity by different professional groups and utility in terms of usability, time to complete and ease of interpretation. Barriers to use of the ART identified during the pilot trial were the time required to complete, changes required to local systems and procedures and the finite availability of rehabilitation services which were not anticipated to meet the rehabilitation needs (as defined by the ART) of patients with stroke in Australia [16]. Users from all pilot sites reported that training in the form of education and educational resources would be required for a national roll-out of the ART (S. Hillier, personal communication, April 2012)

In December 2012, the final version of the ART was passively disseminated via email to stroke clinicians working in Australian hospitals and to Stroke Clinical Networks in all Australian states. Since then, the ART and related educational resources can be downloaded for free from the Australian Stroke Coalition website as part of the dissemination strategy (available from http://australianstrokecoalition.com.au/projects/assessment-for-rehabilitation-pathway-and-decision-making-toolnual-and-decision-making-tool/).

Passive dissemination of a resource such as the ART does not ensure its use. Clinicians must first know about the resource, access the website, download and print copies of the ART and then determine how to use the ART in clinical practice. When clinicians are provided with printed educational materials, processes of care tend to improve slightly [17, 18]. Two systematic reviews have reported on direct comparisons of passive dissemination of printed educational materials (such as the ART) to strategies that include face-to-face education sessions. Both reviews provided evidence that the education sessions were more effective for changing health professionals’ behaviour than the passive techniques [17, 19].

Multifaceted interventions to change practice are interventions comprised of two or more components [20]. A cluster randomised controlled trial conducted after the aforementioned systematic reviews were published, provided evidence that a multifaceted intervention was more effective than passive guideline dissemination alone for improving process of care and patient outcomes in Australian acute stroke units [21, 22]. However, very little is known about the relative effectiveness of multifaceted interventions compared to education interventions (with or without printed educational materials), particularly when targeting multidisciplinary teams working in acute hospitals. The most recent Cochrane review regarding the effectiveness of onsite education interventions included 69 studies [19], but only five studies compared education interventions (with or without printed educational materials) to a multifaceted intervention [23–27]. None of these studies involved multidisciplinary teams within hospitals. No recent literature was identified which compared education to multifaceted interventions for professional behaviour change in any healthcare setting.

This study was designed to investigate and compare two implementation interventions (education-only or a multifaceted intervention) for improving rehabilitation assessment practices for patients with stroke in Australian hospitals by acute hospital clinicians. Data from the national audit provided information on rehabilitation assessment practices in hospitals not participating in the trial. To avoid contamination, a cluster design was used, with the hospital as the unit of randomisation.

The primary research question was

Is a multifaceted intervention more effective than an education-only intervention for increasing the proportion of patients with stroke who are assessed for the need for rehabilitation by hospital clinicians?

The secondary research question was

Does an education-only or a multifaceted intervention ensure that recommended (ART) criteria are used when patients with stroke are assessed by acute hospital clinicians and reported to have no rehabilitation needs?

## Methods

A cluster randomised trial design was used, with hospitals randomly allocated to receive either an education-only intervention or a multifaceted intervention. The outcome of interest was documentation of rehabilitation needs in the medical records of patients with stroke by a health professional providing ward-based care in the acute hospital. The trial registration number is ACTRN12616000340437.

All hospitals in South Australia (SA) with organised stroke services were eligible to participate in the trial. Hospitals in other states of Australia with acute stroke units, admitting more than 100 patients with stroke were also eligible. An invitation to participate in a trial of the ART was sent by the Australian Stroke Coalition to hospital clinicians, administrators and stroke networks around Australia. In South Australia, senior hospital-based clinical staff were invited to participate in the trial by the South Australian Stroke Clinical Network.

Recruited hospitals were stratified by state, region (metropolitan, regional) and the proportion of patients that had their rehabilitation needs assessed in the 2011 national audit. After stratification, hospitals were randomly assigned to receive either an education-only intervention or a multifaceted intervention. The randomisation schedule was generated by computer program (https://www.sealedenvelope.com/simple-randomiser/v1/lists) on 19/3/2013 by a third party, blind to the specific hospital list. Allocation was then undertaken by assigning the coded hospitals to the list based on the stratification.

### The implementation interventions

A rolling recruitment and intervention delivery strategy was used, and the interventions were commenced at the different sites over a 14-month period (first site commenced April 2013; tenth site commenced June 2014). All interventions were delivered by author EL. The TIDieR checklist was used to guide reporting [28] (Additional file 1). All medical, allied health and senior nursing staff working on the acute stroke unit (metropolitan hospitals) or in the rehabilitation team (regional hospitals) were invited via email to participate in the interventions. Participation in the interventions was not mandated at any site.

The education-only intervention consisted of a single onsite education session and distribution of printed copies of the ART and the ART user manual. Education sessions were conducted in rooms on or near the stroke unit or the rehabilitation departments at the regional hospitals. Participants (and EL who delivered the intervention) were seated, at most sites around a table. Information was provided verbally about why the ART was developed and how it was designed to be used. It was emphasised that while use of the ART might not change access to the currently available rehabilitation services, use of the ART would quantify whether there were sufficient rehabilitation services available to meet the needs of patients with stroke. Copies of the ART and the user manual were distributed, and participants were encouraged to ask questions. Information was provided regarding the online resources. As the trial progressed, participants reported strategies that improved rehabilitation assessment practices to the research team. This information was incorporated into the education sessions for all sites which participated in the interventions at later dates. Education sessions lasted between 30 and 60 min.

**Fig. 1:**
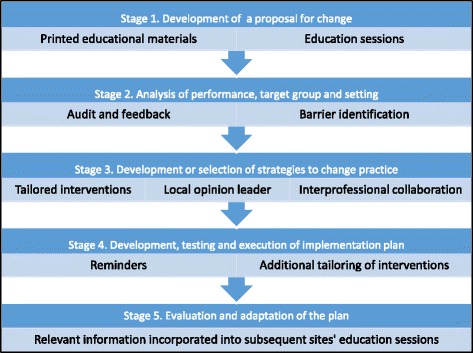
Overview of implementation of change model and selected implementation strategies

The multifaceted intervention was developed using the implementation of change theoretical model [29] which has been used extensively in the implementation of new guidelines and best practices and procedures [30]. Figure 1 illustrates the theoretical model and the selected strategies of the multifaceted intervention.

The multifaceted intervention was spaced over 1 to 2 weeks and included two onsite education sessions [19, 31] and distribution of the ART [18]. Opinion leaders [32] and reminders [17] have been shown to increase the effectiveness of education interventions, so these strategies were incorporated into the intervention. Audit and feedback [12] was included to provide information to participants about their site’s performance. A barrier identification and local strategy development session [33] was facilitated by author EL. Interdisciplinary team work [34] and development of time-efficient systems and procedures were promoted during the strategy development discussions.

The second education sessions and other strategies involved in the multifaceted intervention occurred approximately 1 week after the first education session. Education sessions were co-presented by a member of the local Stroke Clinical Network. Site-specific feedback from the pre-intervention medical record audit was provided as part of the second education session, which lasted between 30 and 60 min. Feedback was presented verbally and in writing by author EL regarding proportions of patients assessed for rehabilitation, details of people who conducted the assessments and demographic and stroke details of patients who were not assessed.

A 1-h workshop was held in the same week as the second education session to identify barriers and develop local strategies to support use of the ART. Workshop participants were asked to brainstorm factors that might make using the ART with every patient difficult, and these barriers were written onto a whiteboard or butcher’s paper. When participants and the facilitator were satisfied that all potential barriers had been listed, each participant wrote on a piece of paper the three most important barriers to address during the strategy development session. Responses were tallied to identify the most commonly nominated barriers. The rest of the session (approximately 30 min) was dedicated to developing strategies to address these barriers. The facilitator encouraged development of strategies wherein responsibility was shared by more than one member of the team (in line with recommendations from the ART pilot trial) and strategies which reduced redundancy of paperwork.

Site champions volunteered or were nominated by other participants to assume primary responsibility for executing the implementation plan, with support from other team members. Reminders in the form of audit feedback, workshop minutes and action plans were sent to all participants. Site champions were contacted by email or telephone everyone 1 or 2 weeks in the month following the intervention. Details of the education-only intervention and multifaceted intervention are provided in Table 1.

**Table 1:** Details of the implementation interventions

Education-only intervention: single onsite education session and provision of printed educational resources	
• Presented at each site by author/researcher EL • Lasted 30 min • Information provided regarding rationale for ART development, how use of ART complies with local stroke pathways, how ART was used at other sites • Provision of hard copies of ART, details where to access online resources	
Multifaceted intervention	
• Presented at each site by author/researcher EL • Education intervention as above, co-presented by member of local stroke network (30 min) • Verbal feedback from medical record audit (30 min), written feedback distributed to all participants • Facilitated workshop (60 min) to identify site-specific barriers to use of ART, identify and tailor strategies to local barriers. Facilitator encouraged development of strategies to enhance collaboration between team members and reduce duplication of assessments and paperwork • Site champion self-nominated at each site to lead use of ART • Reminder emails (minutes of strategy development session, proposed actions) sent to all participants, phone calls and emails to site champion +/− site visits • Strategies nominated by participants which required input from research team o Provision of up to 3 extra education sessions (sites 1 and 3) o Organisation of hospital-approved ART paperwork (sites 1, 3, 10) o Provision of further details from medical record audit (Site 3) o Attendance at team meeting to support use of ART when first introduced (site 1)	

At the conclusion of the onsite intervention session(s), participants were asked as a group to use the ART with every patient with stroke; this entailed the team documenting the patient’s current level of function on each of the 14 domains and referring all patients to a rehabilitation service unless they met the ART exception criteria. A 4-month implementation period was selected for all sites to execute their chosen strategies for implementing the ART and improving rehabilitation assessment practices. Participants at sites assigned to the education-only intervention were requested not to discuss use of the ART with colleagues from other participating hospitals.

### Data collection

#### Medical record audit

Retrospective medical record audits were conducted by two registered physiotherapists who were blinded to hospital allocation both before and 6 months after the implementation interventions using identical procedures. Author EL conducted all pre-intervention audits prior to being told hospital allocations. A research assistant was employed and trained by EL to conduct all post-intervention audits. Both people independently audited the medical records of 32 patients. Inter-rater reliability of the main outcome (assessment for rehabilitation by hospital clinician) was excellent with *k* value of 0.87 (agreement 94 %). Differences in results were discussed to ensure even greater calibration was achieved for the ongoing collection of data.

The pre-intervention cohort consisted of patients with a diagnosis of acute stroke who were discharged from hospital consecutively from a set date 3 months prior to the intervention commencing at each site—due to the rolling intervention delivery strategy used, pre-intervention audits at the 10 hospitals were sequentially conducted over a 15-month period commencing in December 2012. Patients in the post-intervention cohort were discharged from hospital consecutively from a set date 4 months after the last onsite intervention session. Data were collected regarding admitting hospital, patient age, gender, pre-stroke living status (community or residential care, home alone or with others), stroke severity on admission (National Institute of Health Stroke Severity Scale), stroke type (Oxfordshire Stroke Classification) and discharge destination. A minimum of 32 and maximum of 45 medical records were audited at each metropolitan site at the two time points. Fewer medical records were available at the regional hospitals, so all available medical records that were identified within the pre-specified time-frame were included in the audit.

The primary outcome was whether a patient’s rehabilitation needs were assessed by a health professional providing ward-based care; this was defined as documentation regarding the patients’ suitability for rehabilitation, rehabilitation requirements or potential to improve with further therapy or rehabilitation. Assessment details were collected and included the discipline of the person who conducted the assessment and whether rehabilitation was recommended. When rehabilitation was not recommended, the reason given by the assessor(s) was recorded. Patient records were excluded from further analysis if rehabilitation assessments were not indicated (patients were receiving palliative care or the patient was in a coma) [15].

### Statistical analysis

Audit data analysis was performed in STATA version 14 [35]. A power calculation was performed using an anticipated moderate effect size (*d* = 0.36) in the group assigned to the multifaceted intervention, based on recent results from Australian acute stroke units [22]. With alpha of 5 % and power of 80 %, taking into consideration the clustering effect for the 10 hospitals which on advice was set as “low” (intracluster correlation = 0.01), the required sample size of reviewed medical records of patients with stroke was calculated to be 310 across the 10 sites at each time point.

Descriptive analysis was used to determine the frequency of rehabilitation assessments by hospital clinicians. The reasons given when rehabilitation was not recommended were tallied. The change over time in the proportion of patients assessed for rehabilitation was analysed using chi-squared tests.

The difference in the proportion of patients whose rehabilitation needs were assessed by hospital clinicians before and after the intervention was also analysed using mixed-effects logistic regression analysis, with time period as the dependent variable and hospital included as the random effect to account for correlations between patients within individual hospitals. This different was investigated in the overall sample and for each intervention type separately. In order to test for differences between the two interventions, an interaction term between the intervention type and time period was included as the dependent variable in the same model. Output was reported as odds ratio with 95 % confidence intervals. The significance threshold was set at 0.05.

## Results

**Fig. 2:**
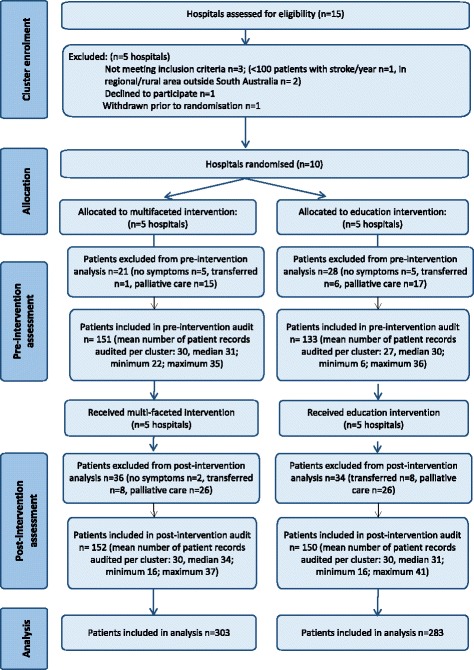
CONSORT flow diagram

An overview of the results from the trial is presented using a CONSORT flow diagram for cluster randomised trials (Fig. 2).

Ten hospitals in two states of Australia (SA and New South Wales, NSW) were recruited. All hospitals in SA with organised stroke services (2 regional hospitals without acute stroke units, 4 metropolitan hospitals with acute stroke units) participated. Four metropolitan hospitals in NSW were also recruited. Details of the participating hospitals are provided in Table 2. Metropolitan hospitals with acute stroke units were assigned numbers 1 to 8, and the two regional hospitals were assigned numbers 9 and 10. No participating site was using the ART prior to the study.

**Table 2:** Details of participating hospitals

Site	State	Allocated intervention	Acute stroke unit beds	Patients with stroke admitted each year	
				To hospital	To acute stroke unit
1	South Australia	Multifaceted	14	800	580
2	South Australia	Education	20	800	800
3	South Australia	Multifaceted	6	350	350
4	South Australia	Education	12	300	270
5	New South Wales	Education	4	130–150	130–150
6	New South Wales	Multifaceted	4	300–400	280
7	New South Wales	Multifaceted	4	350	250
8	New South Wales	Education	30^a^	500	400
9	South Australia	Multifaceted	0	30	Not applicable
10	South Australia	Education	0	30	Not applicable

^a^Stroke beds not funded separately from neurological beds, 30 beds on the stroke and neurological ward

Medical records of 333 patients were audited prior to the intervention and 372 patients post-intervention. Data from 49 patients in the pre-intervention cohort and 70 patients from the post-intervention cohort were subsequently excluded as they had no symptoms of stroke on admission, were transferred to another hospital for ongoing investigation or medical management or received palliative care. Therefore, data from 586 patients were included in the statistical analysis (post-intervention *n* = 302; multi-faceted intervention *n* = 152, education intervention *n* = 150). These figures resulted in an overall power of 79 % in the post-intervention cohort. Rehabilitation assessment practices prior to the interventions have been previously reported [36].

Data regarding the intervention participants, numbers of intervention sessions and details of the self-nominated site champions are presented in Table 3. The majority of participants were allied health and nursing staff. Medical staff from the acute stroke unit attended interventions at only two sites. Interventions were delivered as intended at all sites with the exceptions of sites 3 and 7 (both assigned to multifaceted intervention) where the strategy development workshop was compressed into 30 min rather than the originally anticipated 60 min. Participants at site 7 requested to have the second education and audit feedback session combined with the strategy development workshop into a single 1-h session. At site 3, the strategy development workshop was attended by a rehabilitation physician who had not attended the previous education sessions and requested extensive clarification about the ART and the associated research project. Following the provision of this information, only 30 min was available to identify barriers and develop strategies to change rehabilitation assessment practices. Due to the clinical demands of the participants, the research team and the site champion were unable to schedule a further strategy development workshop.

**Table 3:** Details of participants in the implementation interventions

Site	Site champion	Attendees		
		Education session	Education + audit feedback	Strategy development
1	Nurse	Nurse × 2 Physiotherapist × 2 Occupational therapist × 2 Speech pathologist × 2 Dietician Social worker Stroke network representative (*n* = 10) *Extra session:* Physiotherapy department (*n* = 30)	Medical intern Nurse × 2 Physiotherapist × 2 Occupational therapist × 2 Speech pathologist × 2 Dietician Social worker STROKE network representative (*n* = 11)	Medical resident Nurse Physiotherapist Occupational therapist Speech pathologist Dietician Social worker Stroke network representative (*n* = 7)
2	N/A	Physiotherapist Occupational therapist Speech pathologist Dietician Social worker (*n* = 5)	N/A	N/A
3	Nurse	Medical resident Nurse × 2 Physiotherapist × 2 Occupational therapist × 2 Speech pathologist × 2 Dietician Rehabilitation nurse Stroke network representative (*n* = 11) *Extra session:* nurse × 6	Medical resident Nurse × 2 Physiotherapist Occupational therapist Rehabilitation nurse Stroke network representative (*n* = 6)	Nurse Physiotherapist Occupational therapist Speech pathologist Dietician Social worker Rehabilitation physician Rehabilitation nurse Stroke network representative (*n* = 8)
4	N/A	Nurse Physiotherapist Occupational therapist Speech pathologist Social worker Rehabilitation physician Rehabilitation nurse (*n* = 7)	N/A	N/A
5	N/A	Nurse Physiotherapist Occupational therapist Speech pathologist Social worker (*n* = 5)	N/A	N/A
6	Speech pathologist	Nurse × 2 Physiotherapist Occupational therapist Speech pathologist Dietician Social worker (*n* = 7)	Nurse × 2 Physiotherapist Occupational therapist Speech pathologist Dietician Stroke network representative (*n* = 6)	Nurse × 2 Physiotherapist Occupational therapist × 2 Speech pathologist Stroke network representative (*n* = 6)
7	Nurse	Nurse Physiotherapist Occupational therapist Speech pathologist Social worker Rehabilitation physician (*n* = 6)	*Held concurrently at participants request* Nurse Physiotherapist Speech pathologist Social worker Stroke network representative (*n* = 4)	
8	N/A	Nurse × 2 Physiotherapist × 2 Occupational therapist (*n* = 5)	N/A	N/A
9	Nurse	Nurse × 3 Physiotherapist × 2 Occupational therapist Speech pathologist Allied health assistant (*n* = 8)	Nurse × 3 Physiotherapist × 2 Occupational therapist Speech pathologist Allied health assistant (*n* = 8)	Nurse Physiotherapist Visiting medical registrar (*n* = 3)
10	N/A	Nurse × 2 Physiotherapist Occupational therapist Speech pathologist (*n* = 5)	N/A	N/A

*N/A* hospitals assigned to education intervention so did not nominate a site champion or participate in audit feedback or strategy development sessions

Strategies developed to support use of the ART commonly involved adaptation of team meeting documents, creating action plans regarding when and by whom the ART would be completed, and how information from the ART would be used. Other strategies were additional education sessions (sites 1 and 3), further information from the medical record audit (site 3) and support from the research team when the ART was first used (onsite support during team meeting at site 1; advice over the telephone sites 6 and 9).

All education sessions and workshops were delivered by one person (author EL), thus ensuring consistency of information delivery and intervention processes. The presence of Stroke Clinical Network representatives at interventions at sites assigned to the multifaceted intervention confirmed that the information being presented was accurate and appropriate for the different state contexts. In follow-up focus groups (reported elsewhere) [37], participants at all sites reported they did not participate in any other activities about the ART beyond those organised with, or communicated to, the research team.

Data regarding patient demographics, stroke type and stroke severity of the post-intervention cohort are presented in Table 4. Age, gender, pre-stroke mobility, pre-stroke living status, stroke type and stroke severity were similar for patients in the multifaceted intervention and education groups.

**Table 4:** Demographic and stroke-related details of patients included in post-intervention audit

Patient factor	Multifaceted intervention (*n* = 152)	Education intervention (*n* = 150)	*p* value**
Age^a^	Median 77 (min 30, max 97)	Median 78 (min 15, max 96)	0.75 (*t* test)
Male^a^	86 (57 %)	84 (57 %)	0.98
Independently mobile pre-stroke^a^	139 (92 %)	144 (96 %)	0.22
Born in Australia^a^	76 (52 %)	72 (50 %)	0.77
Pre-stroke living status^a^			0.19
Home with others Home alone Residential care	91 (60 %) 38 (25 %) 22 (15 %)	95 (63 %) 43 (29 %) 12 (8 %)	
National Institutes of Health Stroke Scale on admission			0.45
8< 8–16 >16	117 (77 %) 28 (18 %) 7 (5 %)	118 (79 %) 29 (19 %) 3 (2 %)	
Oxfordshire Stroke Classification			0.15
Total anterior circulation infarct Partial anterior circulation infarct Posterior circulation infarct Lacunar infarct Intracerebral haemorrhage	13 (9 %) 46 (30 %) 23 (15 %) 58 (38 %) 12 (8 %)	8 (5 %) 39 (26 %) 28 (19 %) 51 (34 %) 24 (16 %)	

**Chi-squared test unless stated

^a^Data missing: gender *n* = 2, age *n* = 2, pre-stroke living status *n* = 1, independently mobile pre-stroke *n* = 1, born in Australia *n* = 11

### Effectiveness of a multifaceted intervention versus an education-only intervention for improving the proportion of patients with stroke who are assessed for rehabilitation

In unvariable analyses, the proportion of patients assessed for rehabilitation by hospital clinicians increased (post-intervention versus pre-intervention) at all participating sites, although the increase was not always statistically (see Table 5).

**Table 5:** Rehabilitation assessments by hospital clinicians pre- and post-intervention

Site	Pre-intervention (*N* = 284)	Post-intervention (*N* = 302)	Chi-squared test *p* value	Odds ratio (95 % confidence interval), *p* value
Multifaceted intervention				
Site 1	18/35 (51 %)	23/37 (62 %)	0.36	
Site 3	12/35 (34 %)	25/30 (83 %)	<0.001	
Site 6	12/28 (43 %)	28/34 (82 %)	0.001	
Site 7	18/31 (58 %)	23/35 (66 %)	0.52	
Site 9	1/22 (4 %)	13/16 (81 %)	<0.001	
Total multifaceted intervention	61/151 (40 %)	112/152 (74 %)	<0.001	4.13 (2.54–6.71), <0.001
Education intervention				
Site 2	22/36 (61 %)	24/29 (83 %)	0.06	
Site 4	20/35 (57 %)	26/31 (84 %)	0.02	
Site 5	12/30 (40 %)	21/33 (64 %)	0.06	
Site 8	7/26 (27 %)	26/41 (63 %)	0.004	
Site 10	0/6 (0 %)	5/16 (31 %)	0.12	
Total education intervention	61/133 (46 %)	102/150 (68 %)	<0.001	3.41 (1.99–5.84), <0.001
Total of whole sample	122/284 (43 %)	214/302 (71 %)	<0.001	3.69 (2.57–5.30), <0.001

Following adjustment for time and correlation of patients within hospitals using the random-effects statistical analysis method, there was no difference between the multifaceted intervention and the education-only intervention in terms of the odds of a patient being assessed for rehabilitation by a hospital clinician (reference category education-only intervention; odds ratio 1.29, 95 % confidence interval 0.63–2.67, *p* = 0.483).

Regardless of intervention received, the odds of a patient receiving an assessment for rehabilitation in the post-intervention period was 3.69 times greater compared to pre-intervention (95 % confidence interval 2.57–5.30, *p* < 0.001).

### Use of recommended (ART) criteria when patients were assessed and not recommended for rehabilitation

Results of the post-intervention audit indicated that 214 patients were assessed for rehabilitation by a hospital clinician. The majority (*n* = 175, 82 %) of patients assessed were reported to need rehabilitation. Assessments of the 38 patients who were deemed to not need rehabilitation were examined. Details of these assessments are presented in Table 6. ART criteria were not used in 17 (45 %) of the documented rehabilitation assessments which did not identify rehabilitation needs. The type of intervention received did not appear to influence use of the ART criteria when assessing patients with stroke for rehabilitation.

**Table 6:** Reasons rehabilitation was not recommended for patients in the post-intervention audit

Reason rehabilitation not recommended by hospital clinician (*n* = 38)	Multifaceted intervention (*n* = 27)	Education intervention (*n* = 11)
Assessment for Rehabilitation Tool criteria (*n* = 21)		
Fully recovered	15	5
Patient or family refused	1	0
Other reasons documented (*n* = 17)		
For residential care placement	1	2
Poor motivation/participation	1	2
Unwell, medically unstable	1	1
Not following instructions	2	0
Other, or reason not given	6	1

## Discussion

The main aim of this study was to compare the effectiveness of two implementation interventions for improving rehabilitation assessment practices by health professionals working with patients with stroke. This study is the first to our knowledge that has been used to compare the relative effectiveness of an education-only intervention to a multifaceted intervention for healthcare teams working in hospital settings. The intervention received did not significantly affect the proportion of patients who had their rehabilitation needs assessed. However, both the education intervention and the multifaceted intervention appeared to influence clinical practice; the proportion of patients assessed by hospital clinicians in the overall sample increased by 28 %. In contrast, the proportion of patients assessed for rehabilitation improved in Australian hospitals not involved in the trial by 4 % following the release of the ART (from 45 % in 2011 to 49 % in 2013) [National Stroke Foundation, unpublished data]. Therefore, although a control group was not included in the study, the two implementation interventions appeared to be more effective than passive dissemination of the ART resources alone. Our results compare favourably with median results of 6 % that were reported in a systematic review regarding the effect of onsite education interventions on professional practice and health care outcomes [17, 19].

In previous research, multifaceted interventions in Australian acute stroke units were significantly more effective than guideline distribution alone for improving processes of care and outcomes for patients with stroke [21, 22]. We hypothesised that a multifaceted intervention would be more effective than a single education-only intervention for improving proportions of patients who were assessed for rehabilitation. However, the addition of audit and feedback, tailored interventions, recruitment of site champions, reminders and interdisciplinary teamwork did not augment the effectiveness of the education intervention. While cost of the interventions was not formally evaluated, it is likely that a single education intervention would have important cost savings compared to the equally effective but more time-intensive multifaceted intervention.

Audit and feedback is most effective when baseline performance is poor [12, 38], whereas three of the sites assigned to the multifaceted intervention were audited at baseline as documenting rehabilitation assessments for a similar or greater proportion of patients than the national average. In line with recommendations, audit and feedback was provided verbally and in writing, and action plans were organised via the strategy development workshop (discussed below). However, due to pragmatics of the trial, some recommended audit and feedback practices could not be performed, such as the audit being conducted by a respected colleague rather than an external researcher, and being provided on more than one occasion [12, 38].

The tailored interventions were selected pragmatically by the participants according to their preferences, and mostly consisted of creating action plans to change rehabilitation assessment practices, creating or adapting documents to support these changes, and organising extra education sessions about the ART. Two sites which were assigned to the education-only intervention also reported using some of these same strategies. It could be considered that the format of the education sessions was a source of contamination between the groups, because information arising from early sites regarding use of the ART was incorporated into education sessions for all participating sites. The first site to initiate changes to the case conference document was a site which received the education-only intervention, and this strategy was subsequently discussed at other education sessions in the study and subsequently used at two sites assigned to the multifaceted intervention.

Reminders are most likely to be successful at changing practice when they meet the specific need of the intended users and proactively prompt clinicians to perform certain behaviours [17]. Therefore, in the context of the current research, the reminders initiated by the research team (emails, phone calls or site visits) may not have prompted the participants to use the ART as these reminders were generally received at times when the participants were not providing patient care or attending case conference meetings. In contrast, the strategy developed at three hospitals (one education-only, two multifaceted intervention) of adding a check-box regarding a rehabilitation assessment recommendation to the case conference document was a form of participant-initiated reminders that prompted staff to discuss and document an assessment as part of the case conference discussion.

Opinion leaders are people who through their interpersonal skills, leadership abilities and positioning within the communication structures of the workplace are able to influence change in workplace behaviours through informal methods [32, 39]. There was a participant at every site, whether assigned to receive the education-only or multifaceted intervention, who appeared to be an informal opinion leader (using informal observation methods during the intervention sessions and post-intervention evaluation sessions). In most cases, these clinicians appeared to support the use of the ART without further encouragement or support from the research team. Therefore, the intervention of formally recruiting a clinician as an ART champion or ‘opinion leader’ may have been no more effective than the education-only intervention because the majority of the participating clinicians (and informal opinion leaders) by choosing to participate in the research trial were already motivated to improve clinical practice and were able to implement change successfully after being educated about the ART.

Despite a significantly larger proportion of patients being assessed for rehabilitation in the post-intervention period, more than one quarter of patients included in the post-intervention medical record audit still did not have a documented rehabilitation assessment. Overall, neither intervention appeared to be effective in addressing *how* clinicians assessed or referred patients for rehabilitation because the majority of sites did not consistently recommend rehabilitation for all patients who did not meet the ART exception criteria. The factors that influenced whether participants changed the way they assessed patients’ rehabilitation needs and how they referred patients to rehabilitation were identified and described in a separate qualitative study embedded within this cluster randomised trial [37].

It has been suggested that there may not be adequate rehabilitation resources in Australia to meet the needs of every patient with stroke [9, 14]. However, unmet rehabilitation needs have not been identified in medical record audits of patients with stroke in Australian hospitals [36]. In earlier work, some participants in our study reported that the availability of rehabilitation services was a factor they considered when deciding whether to recommend ongoing rehabilitation for patients [40]. Participants at all sites expressed the opinion that there would not be enough rehabilitation resources to meet the requirements of all people who had rehabilitation needs identified through use of the ART [37]. This perceived shortage of rehabilitation service availability influenced participants at some sites not to refer all people with identified rehabilitation requirements to a rehabilitation service [37]. We argue that in order to better understand current rehabilitation requirements and ensure equity of access to rehabilitation after stroke, it is important that consistent criteria are used when assessing patients’ rehabilitation needs and when referring patients to rehabilitation services. Further work is required to achieve this standard for every patient with stroke in Australia.

The strengths of this study were that a structured framework was used to guide the implementation intervention, the consort statement for cluster randomised trials was used to plan the design and to guide the reporting of the trial and the TIDIeR checklist was used to guide reporting of the interventions.

A limitation to the study was that the implementation interventions had negligible reach with medical staff. Medical directors at every site provided consent that the trial could proceed, but medical staff providing ward-based care attended intervention sessions at only 3 of the 10 participating sites. Reasons were not sought when clinicians chose not to attend the intervention sessions. Medical staff have previously been reported to have low levels of engagement in quality improvement initiatives in Australian ASUs [41]. There is evidence that medical professionals respond differently from other health professionals to implementation interventions [42]. Strategies specifically tailored to medical professionals may have enhanced participation of this group.

Qualitative work conducted alongside this study indicated that participants’ relationships with patients, medical staff and rehabilitation service providers influenced whether they changed rehabilitation assessment and referral practices [37]. Recommendations for future work are that other key stakeholder groups (hospital administrators, rehabilitation service providers, healthcare consumers) are involved in the implementation program, to develop a shared understanding of the importance of using evidence-based criteria to assess patients’ rehabilitation needs. An integrated intervention, supported by multiple stakeholder groups, could lead to further improvements in rehabilitation assessment practices. This in turn would lead to better understand rehabilitation requirements for patients with stroke and facilitate the development of systems to ensure equitable access to rehabilitation services.

## Conclusions

This study has provided evidence that a single education-only intervention and provision of printed materials regarding the ART was as effective as a multifaceted implementation intervention for improving proportions of patients with stroke who had their rehabilitation needs assessed. Comparing these results to data from the national audit over the time frame in which the ART was released, the two implementation interventions appeared to be more effective than passive dissemination of the resources alone. Further work is required to ensure that the rehabilitation needs of all patients with stroke are assessed using evidence-based criteria to ensure accurate data are collected regarding the rehabilitation needs of people with stroke in Australia.

## Additional file

Additional file 1: Cluster RCT_TIDieR-Checklist-Word. (DOCX 28 kb)

## Abbreviations

ART: Assessment for Rehabilitation Pathway and Decision-Making Tool
NSW: New South Wales
SA: South Australia
